# Developing a Vaccine Against Human Cytomegalovirus: Identifying and Targeting HCMV’s Immunological Achilles’ Heel

**DOI:** 10.3390/vaccines13050435

**Published:** 2025-04-22

**Authors:** Anastasia Lankina, Marta Raposo, Alexander Hargreaves, Claire Atkinson, Paul Griffiths, Matthew B. Reeves

**Affiliations:** 1 Institute of Immunity and Transplantation, Division of Infection and Immunity, University College London, London NW3 2PP, UK; anastasia.lankina.20@ucl.ac.uk (A.L.); marta.raposo.21@alumni.ucl.ac.uk (M.R.); alexander.hargreaves.23@ucl.ac.uk (A.H.); p.griffiths@ucl.ac.uk (P.G.); 2 School of Applied and Health Sciences, London South Bank University, London SE1 0AA, UK; claire.atkinson@lsbu.ac.uk

**Keywords:** HCMV, virus, glycoprotein, mRNA, transplant

## Abstract

Human cytomegalovirus (HCMV) is a critical pathogen in immunocompromised populations, such as organ transplant recipients as well as congenitally infected neonates with immature immune systems. Despite decades of research and the growing financial burden associated with the management of HCMV, there is no licensed vaccine to date. In this review, we aim to outline the complexity of HCMV and the antigens it presents and the journey and challenges of developing an effective HCMV vaccine, as well as further highlight the recent analyses of the most successful vaccine candidate so far—gB/MF59.

## 1. Clinical Burden of HCMV and the Need for Vaccine Development

Human cytomegalovirus (HCMV) is a globally prevalent herpesvirus, infecting from 60% of the adult population in developed countries to more than 90% in developing countries [1,2]. Although normally asymptomatic in immunocompetent hosts, HCMV can pose a severe threat to immunosuppressed individuals, like those with primary and acquired immunodeficiencies, organ transplant recipients, and immunologically immature foetuses [3]. When not controlled by a robust immune response, HCMV is known to cause mild respiratory and mononucleosis-like illness, which, over time, develops into a life-threatening collection of symptoms known as “HCMV disease” [4].

Unsurprisingly, given the incidence and seroprevalence of HCMV infection, HCMV disease is a common and historically well-known complication in AIDS patients, often manifesting as retinitis, gastrointestinal disease, and other types of end-organ disease [5]. Similarly, HCMV infection is a well-characterised contributor to disease during the immune suppression following solid organ transplantation (SOT) and haematopoietic stem cell transplantation (HSCT) [6,7]. HCMV disease in SOT patients predominantly arises 30–90 days after transplant and, rarely, 180 days after transplant. Starting with a non-descript viral syndrome disease, in severe cases, the disease can escalate with wider organ involvement including in the upper digestive system, viral hepatitis, meningoencephalitis, pancreatitis, and myocarditis. However, unlike for HIV patients, it is rare in HCMV disease for SOT patients to develop retinitis [8,9].

Critically, solid-organ recipients with a pre-existing HCMV immunity (recipient seropositive, R+) develop fewer HCMV-associated complications and less end-organ disease than their HCMV-naive counterparts (recipient seronegative, R−) when transplanted with an infected organ (donor seropositive, D+) [10,11], which suggests that residual HCMV immunity, despite immune suppression, can be protective. Importantly, from a clinical perspective, HCMV infection post-transplant is typically associated with a longer hospital stay and higher associated management costs, higher rates of graft-versus-host disease, and shorter survival among the transplant recipients [12].

Moreover, congenital infection with HCMV (cCMV) is a leading cause of newborn and child morbidity and is associated with hearing impairment, developmental delays, and possible disability later in life [13]. The mother-to-foetus transmission of HCMV can be influenced by both primary viral acquisition by a seronegative mother (maternal primary infection, MPI) and a non-primary reinfection or a reactivation (non-primary infection, NPI) of a cognate virus in a seropositive mother [14]. Interestingly, cCMV prevalence is higher in developing countries (up to 2%) than in developed ones (0.5–0.7%), which mirrors the respective difference in HCMV seroprevalence amongst women of reproductive age [15]. Notably, the lower HCMV seroprevalence of women in the Western world has been the major driver of vaccine development, despite a higher prevalence in developing countries, as MPI cases are associated with a much higher risk of HCMV transmission and, consequently, pathology (30–35%) than NPI (1–3%) [16,17,18].

Clearly, pre-existing HCMV immunity is associated with a great reduction in disease across multiple vulnerable populations, which underscores the public health value of an effective HCMV vaccine. Indeed, the need for HCMV vaccine development was declared a top priority in 2000 by the US Institute of Medicine [19]. A major influencing factor was the substantial economic costs associated with HCMV management, as well as the considerable expense of lifelong support of cCMV-affected children. For example, in the UK, it has been calculated that per individual child, the combined costs of receiving a diagnosis and further treatment for cCMV-associated hearing loss were estimated to be around £ 20,000 in 2015 [20]. On a larger scale, it was estimated that in 2016, the UK’s total annual direct and indirect cCMV management costs were at about £ 732 million [21]. Although calculating the precise economic cost is still a major point of discourse [22], it was predicted to be around $3–4 billion in the US [23,24].

Alongside these very well-established sources of HCMV morbidity, HCMV is also speculated to play a role in various additional conditions, including systemic lupus erythematosus, rheumatoid arthritis, atherosclerosis, heart disease, and some cancers such as colon cancer, glioblastoma, and infant leukaemia, which potentially increases HCMV’s financial, and clinical, impact [25,26].

In this review, we do not intend to provide an exhaustive review of all HCMV vaccines, as this has been covered excellently by colleagues in the field—including a very interesting review of a recent NIH meeting involving key stakeholders in the HCMV vaccine field [27]. That said, we will highlight aspects of past and ongoing HCMV vaccination strategies and associated challenges, including a more detailed focus on recent advancements in the analyses of gB/MF59—the most successful vaccine candidate to date—and the implications for HCMV vaccination in general. Due to the nature of gB/MF59 analyses and their focus on antibody responses as a key correlate of vaccine-associated protection, this review is also primarily focused on humoral rather than cellular immunity.

## 2. HCMV Virion Presents Many Possible Vaccine Targets

The goal of vaccination is to educate the adaptive immune response to effectively respond to infection more rapidly. Indeed, many successful vaccines against other pathogens have relied on the induction of robust neutralising antibodies that target key components of the virion to block infection. The HCMV virion and, in particular, the virion envelope contain multiple glycoprotein complexes that underpin the viral ability to establish infection in a wide spectrum of cell types, such as fibroblasts, endothelial, epithelial, smooth muscle, and myeloid-derived cells [28,29]. Indeed, this broad cell tropism likely explains the extensive pathology seen in multiple sites within the body while also allowing the virus to establish lifelong, persistent infections of the host [30].

To facilitate entry into the various cell types, HCMV utilises several glycoproteins, which are presented on the surface of the virion envelope and form several glycoprotein complexes (gC) essential for establishing infection. These complexes are known as gC-I, II, III, and the pentameric complex (PC), and they play critical roles in viral entry into different cell types [31].

The first glycoprotein complex, gC-I, is a homotrimer of glycoprotein B (gB), which plays a pivotal role in all herpesviruses due to its fusogenic function [32]. It facilitates fusion with a target cell membrane by undergoing a dramatic conformational change and is essential for viral entry into all permissive cell types as well as for the cell-associated spread of the virus [33,34]. A more detailed discussion of gB is provided later in the manuscript.

gC-II is the most abundant complex of the HCMV envelope, consisting of glycoproteins M (gM) and N (gN) [35]. The function of the gM/gN dimer is thought to be involved in initiating the first stage of viral entry by adhering to the highly ubiquitous heparan sulphate proteoglycans on the cell surface [36]. In other herpesviruses, both gM and gN homologues are non-essential for in vitro replication; however, gM is essential for the in vitro replication of HCMV [37]. In particular, components of gC-II were outlined to be important for virion assembly. Introducing mutations to the cytoplasmic tail of gM was noted to correlate with a replication-impaired viral phenotype [38]. Moreover, a similar experimental approach to the carboxy-terminal domain of gN outlined the protein’s role in virion egress and envelopment [39].

gC-III, also known as the trimeric complex, is a heterotrimer of glycoproteins H (gH), L (gL), and O (gO). Within this arrangement, gL and gH play crucial roles in activating gB, while gO serves as a co-receptor in the interaction between the trimer and its cellular receptor PDGFR-α [36,40]. Moreover, the trimeric complex is known to mediate the entry of HCMV into fibroblasts [41]. Both gH and gL were shown to be essential for HCMV replication, as respective deletion mutants were non-viable; however, the deletion of gO led to only moderate viral growth defects [42].

Finally, the pentameric complex (PC) also includes gH and gL, together with UL128, UL130, and UL131. This complex is lost in many laboratory-adapted strains, which likely explains their limited cell tropism compared to wild-type HCMV. Similarly to the gC-II trimer complex, PC plays a vital role in engaging with cell-type-specific receptors for the pH-dependent endocytic route of infection, where PC binds neurophilin-2, replacing PDGFRα as the essential receptor required for infection [43]. In addition to the essential receptor binding, HCMV also binds with a wide range of co-receptors which, while not individually essential for infection cumulatively, are important for promoting infection [36]. PC is known to promote the cell-associated spread of the virus by limiting the release of cell-free virus [44,45]. Furthermore, it is becoming increasingly clear that antibodies directed against the PC are incredibly potent at neutralising infection, which likely underpins the inclusion of PC in many of the new vaccines being tested [46].

## 3. The Journey and Challenges of HCMV Vaccine Development

Usually, the goal of immunisation is to elicit a robust immune response similar to the one observed in a natural infection, which is a strategy that has repeatedly proven successful for many pathogens of major medical importance, for example, SARS-CoV-2 during the COVID-19 pandemic [47]. One approach used against several viral pathogens is the application of live attenuated viruses (LAVs) or live modified viruses (LMVs). Historically, the LAV approach was among the first strategies for HCMV vaccine development.

The pioneering publications on the LAV vaccine, based on a culture-passaged clinical strain of HCMV called Towne, reported vaccine efficacy and the ability to elicit neutralising and complement-fixing antibodies, as well as a lack of undesirable side effects [48]. Despite its strong safety profile, the Towne vaccine has demonstrated mixed performance in its numerous clinical trials. While prior immunisation with it was able to provide protection from severe HCMV disease for kidney transplant recipients, it was ineffective at preventing congenital infection [49,50]. Similar results were obtained for an LAV based on four chimeric strains of Towne recombined to contain genomic inclusions from a non-attenuated Toledo strain [51]. The observed lack of functional immunogenicity in the Towne and Towne/Toledo vaccines could have been caused by the extensive attenuation of the virus by culture-passaging and subsequent loss of the expression of antigens like PC, which are important targets for humoral immunity [52].

The lack of efficacy of live vaccines against HCMV outlines one of the many unique challenges associated with HCMV (or any persistent lifelong infection): there is no precedent of normally occurring sterilising immunity that a vaccine could aim to replicate. These observations could be interpreted to suggest that in order to develop a sterilising HCMV vaccine (if sterilising immunity is the desired endpoint), it may not be enough to simply mimic natural infection—instead, the vaccine must be better than nature, either by focusing the broad immune response against specific targets critical for control or inducing responses not normally seen in natural infection.

Indeed, this highlights another significant challenge that became apparent during the journey of HCMV vaccine development: the incomplete understanding of immune correlates of protection within the robust anti-HCMV immune response we observe in natural infection and, by extension, quantifiable targets of a successful vaccine [53]. This is further complicated by data suggesting that these correlates vary amongst different interest groups such as pregnant/postpartum women and transplant patients, which complicates decisions regarding vaccine target groups [54]. Put simply, an immune response that protects in one setting may not be so important in another.

With these ideas to the fore, it is perhaps unsurprising to see that many candidates developed after the Towne and Towne/Toledo vaccines aimed to generate a more robust and targeted immune response by focusing on one or a selected few viral antigens based on our understanding of the immune response to natural infection.

In the interest of space and brevity, in Table 1, we summarise the several HCMV proteins and protein complexes that have been explored as prospective vaccine targets and associated publications. Moreover, we include the different antigen delivery and presentation methods that have been explored and assessed for their efficacy. Due to the nature and focus of this review, we will be further highlighting the gB/MF59 vaccine, which has been demonstrated to be the most successful to date, achieving 43–50% efficacy in phase II clinical trials in women and transplant patients [55,56,57]. Given its apparent superior performance, understanding the protective responses elicited by the gB/MF59 vaccine has come to the fore for advancing HCMV vaccine development and is a major focus of recent HCMV vaccine studies and this review.

## 4. Glycoprotein B as an Immune Target

**Figure 1:**
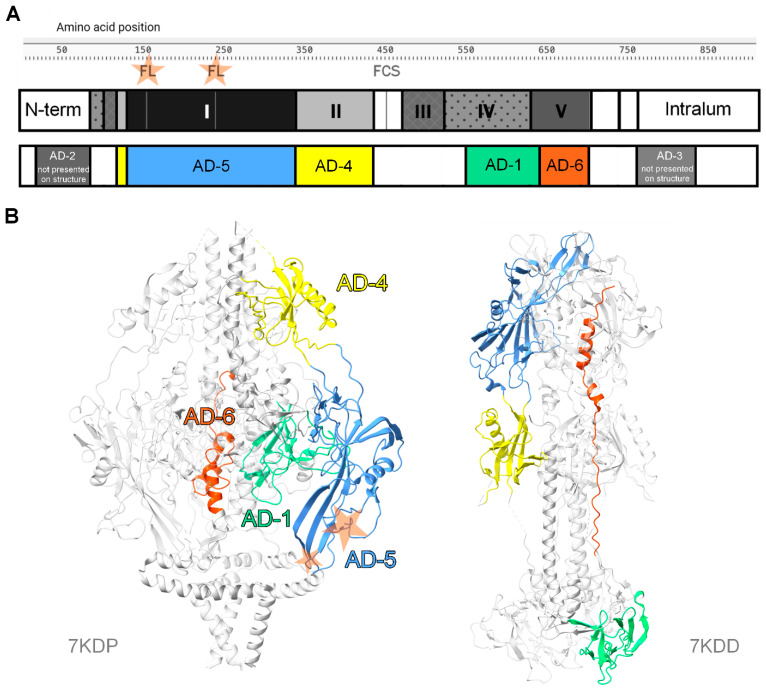
Structural and antigenic domains of HCMV gB pre-fusion (PDB ID 7KDP) and post-fusion (PDB ID 7KDD) trimeric states (**A**,**B**). FL stands for fusion loop (2 per each protomer), FCS stands for furin cleavage site.

Glycoprotein B (gB) is a class III fusion protein essential for viral entry into the target cell, regardless of the cell type or route/mechanism of entry. Thus, inhibiting gB function blocks the imperative step of HCMV entry, which should reduce transmission and associated disease. Therefore, gB presents an attractive vaccine target—as is the case for the fusion protein of multiple pathogens, like hemagglutinin for influenza or spike protein for SARS-CoV-2. Structurally and functionally, gB is conserved across the different HCMV strains and other herpes viruses, and at the sequence level, it maintains a high level of amino acid-level conservation across all HCMVs (96.1%), which adds to its attractiveness as a vaccine target [70]. Consequently, gB immunogenicity and generated antibody responses have been explored extensively, which has culminated in the identification of five conventional and one vaccine-specific antigenic domains within the protein, as shown in Figure 1. Curiously, the nomenclature of antigenic domains, and their being separate entities from the structural domains of gB, is unique to HCMV, with other herpesviruses (e.g., HSV-1 or EBV) relying on the structural or functional regions as descriptors for gB antigenicity [71].

Chronologically, the first identified antigenic domains (AD) were AD-1, AD-2, and AD-3.

Largely overlapping with structural domain IV, AD-1 is located around 550–640aa in the gB linear sequence (Towne strain) and is one of the most immunodominant regions of the protein [72]. Indeed, AD-1 is a well-characterised target for both neutralising and non-neutralising antibodies, with almost 100% of the HCMV-seropositive population having a detectable humoral response towards it [73].

AD-2 is a disordered domain located at the N-terminal end of the protein, consisting of two distinct sites. Site I, located at 64–78aa in Towne, has a sequence that is highly conserved across different strains of the virus. Moreover, AD-2 site I is a well-known target of potent neutralising antibodies [74,75]. Despite that, AD-2 site I is not particularly immunodominant, as it is hypothesised that for a specific response to arise, a very rare VDJ recombination event needs to occur [76,77]. In contrast to the high sequence conservation in site I, the AD-2 site II locus, which is located upstream of site I, exhibits strain-specific variations and also induces antibodies incapable of neutralising the virus in vitro [78]. Interestingly, the neutralising epitope within AD-2 is linear and continuous, which is not typical for a target of a potent neutralising antibody.

The third AD in this list is perhaps the most enigmatic. AD-3 is a highly immunogenic region of gB located in its intraluminal domain, close to the C-terminus of the protein. Despite its consistently high immunogenicity, the antibody response towards AD-3 is exclusively non-neutralising, potentially attributable to its inaccessibility to the surrounding external environment [79]. Thus, the role of AD-3 antibodies in the control of HCMV infection, if any, remains uncertain.

More recently, two additional antigenic domains were discovered: AD-4 and AD-5. Located at 121–132aa and 344–348aa, AD-4 is a conformationally disjointed domain that majorly overlaps with the globular structural domain II [80]. AD-5 is located between the two sections of AD-4, corresponding to the 133–343aa in the linear sequence of gB, and is conformationally concordant with structural domain I. Notably, both AD-4 and AD-5 are known to contain epitopes that are targets of non-neutralising as well as potent neutralising antibodies [81]. Both domains are highly immunogenic, as up to 90% of the seropositive population was reported as displaying neutralising and non-neutralising responses against AD-4 and up to 50% displaying responses against AD-5 [80]. It is hypothesised that antibodies directed against AD-4 and AD-5 likely recognise conformational epitopes due to the high degree of structural information in these regions [82].

For a long time, the five antigenic domains presented above were considered the best possible characterisation of the antigenicity of gB, and it is fair to say that this remains true for natural infection. However, recently, we identified another antigenic domain, AD-6, which is a highly conserved 50 amino acid peptide that can be mapped predominantly onto structural domain V and is discussed in detail later. The response to AD-6 is vaccine-specific and non-neutralising against HCMV virions. However, as also discussed in detail later, it was demonstrated that AD-6 antibodies are able to substantially limit the cell-to-cell spread of the virus [83].

## 5. Successes and Lessons of gB/MF59

**Figure 2:**
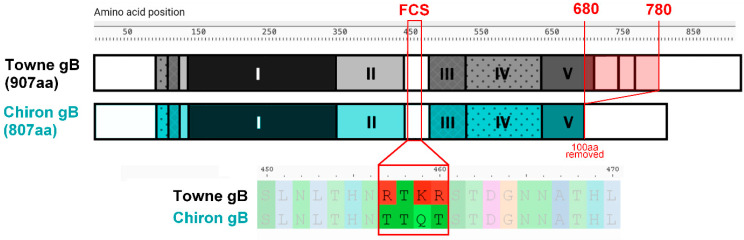
Modifications introduced to the antigen of gB/MF59 (Chiron gB) compared to wild-type gB (Towne, GenBank ID ABQ23592.1). A section of 100 amino acids (680 to 780 in Towne gB) was deleted from the wild-type linear sequence, resulting in the removal of the transmembrane domain and subsequent expression of Chiron gB as a soluble molecule [92].

The identification of AD-6 is the result of substantial interrogation of the humoral immune responses directed against the gB/MF59 vaccine, which is based on a recombinantly expressed soluble modified gB (often referred to as the “Chiron gB”, Figure 2) and an oil-in-water infusion adjuvant MF59 [84,85]. At the timepoint of gB/MF59’s invention, the use of Chiron gB as a vaccine immunogen was a novel approach, while the adjuvant MF59 had already been used in a successful clinical trial of a Novartis influenza vaccine [86]. The first evidence that the gB/MF59 formulation could be a good vaccine construct was supported by exceptional safety and immunogenicity results when administered to the healthy population during the phase I clinical trials, with the resultant antibody titres reportedly exceeding the commercially available HCMV hyperimmune globulin in its neutralising ability [87]. Thus, the vaccine displayed the hallmarks of an excellent vaccine candidate: safety, immunogenicity, and the capacity to induce neutralising antibodies (the longstanding ‘gold standard’ for vaccines directed against fusion proteins).

These promising data in phase I studies led the vaccine to be evaluated in three separate investigator-led phase II clinical trials, all with different target populations of medical importance. Critically, these three trials followed a consistent randomised, double-blind, placebo-controlled methodology, as presented in Table 2, allowing for potential cross-comparison in different target populations.

The first gB/MF59 phase II clinical trial (1999–2009) targeted postpartum women aged 14–40 years [57]. Spanning 42 months, the trial yielded promising results, achieving a 50% reduction in HCMV acquisition: 8% infection in vaccinated individuals versus 14% in placebo. Amongst infants born during the study, there was a trend towards reduced rates of cCMV infection in those born from vaccinated mothers (1% of 81 infants) compared to those born from the placebo group (3% of 97 infants); however, sample sizes were too small for definitive conclusions regarding the prevention of congenital infection. However, these findings outlined the vaccine’s potential to prevent mother-to-foetus transmission, corroborating outcomes from guinea pig studies and a large observational study (3461 participants) achieving the same comparative rates of cCMV infection in infants born to seronegative (3%) versus previously infected mothers (1%) [88,89].

Building on these findings, a subsequent phase II study (2006–2013) began, focusing on adolescent women (12–17 years) and integrating an additional IgG quantification, as well as a comparison of vaccine outcomes to those of natural infection, into its methodology [55]. The vaccine again demonstrated safety and immunogenicity, aligning with the earlier clinical trials. At around 5 months after the second vaccine dose, all gB/MF59-vaccinated individuals had at least a 10-fold increase in gB-specific antibodies, which was further magnified another 10-fold following the third dose and sustained until the final timepoint of the study about 2 years after. These levels surpassed those of placebo participants who became naturally infected during the study, mirroring results from phase I studies in adults [85]. The infection rate was lower in vaccinated patients (6.5%) compared to placebo (11.2%), which translates to a 43% vaccine efficiency, although not statistically significant. This reduced infection rate amongst vaccinees, coupled with higher gB-specific antibody levels in these participants, positioned gB-specific antibodies as a potential correlate of protection against HCMV, requiring further studies to be confirmed.

Finally, a third phase II clinical study (2006–2008) was performed on individuals awaiting a kidney or liver transplant, distinguishing itself from the others by assessing the vaccine’s efficacy in seropositive alongside seronegative patients and measuring the levels of functional neutralising antibodies [56]. The study recorded a substantial increase in the geometric mean titre (GMT) of gB-specific antibodies following the second dose in both seropositive and seronegative vaccine recipients, showcasing an efficiency of gB/MF59 across subpopulations of different immunological status. Interestingly, by day 56 post-vaccination, seropositive patients experienced a significant increase in the titre of neutralising antibodies, correlating with higher gB-specific antibody levels, unlike seronegative patients. This provided the first suggestion that the vaccine’s ability to elicit neutralising antibodies might be influenced by previous viral exposure, highlighting the importance of pre-vaccination serostatus consideration for future vaccination strategies. The magnitude of viraemia post-transplant (experienced by 34.6% of patients) was inversely correlated with the GMT of gB antibodies in both seropositive and seronegative vaccinees. The protective effect was most notably observed in seronegative organ recipients from seropositive donors (the high-risk D+R- group), whereby vaccinated recipients had a shorter duration of viraemia and required less ganciclovir treatment when compared to an equivalent placebo group. This is perhaps not unsurprising—in the R- population (as defined pre-vaccination), the only protection is coming from vaccine-induced immunity, whereas in the R+ population, pre-existing immunity will contribute to the outcomes post-transplant. That caveat aside, these findings confirmed gB-specific antibodies to be a correlate of protection against HCMV viraemia and disease in transplant patients. However, the mechanism by which those antibodies elicit their protection and the reasons for only partial immunity being achieved remained elusive. Notably, gB/MF59 was not progressed to a phase III clinical trial because it did not reach the efficacy threshold predicted to be important for control, and it was not immediately understood how the gB/MF59 vaccine could be improved to achieve that endpoint [90].

It is, of course, important to highlight that using gB as a vaccine target is a concept that has been implemented for multiple vaccine candidates (in various guises), as illustrated by Table 1, so it is intriguing that the gB/MF59 formulation has proven to be particularly successful. This invites the question: what made gB/MF59 so successful? Was it the modified epitope presentation of the recombinant Chiron gB—which, of course, is not equivalent to the complete form of gB presented in the virion? Or, alternatively, the success of gB/MF59, in part, could be linked to the adjuvant used. It is not just the target that is important but also how the immune system is primed to respond to that target, which could have a major impact on the composition of the immune response [91].

Alongside these questions, others were fuelled; for example, do gB-specific antibodies confer protection via direct neutralisation, or are they mediated by non-neutralising antibody responses? How does the humoral response directed against gB/MF59 compare to that in natural infection? These and many more questions laid the foundation for recent research efforts focusing on analyses of the sera collected in all the phase II studies in search of the answers with the aim of understanding and defining the mechanistic correlates of protection of the gB/MF59 vaccine.

## 6. Recent Advances in gB/MF59 Analyses

### 6.1. Neutralising vs. Non-Neutralising Antibodies

Historically, antibodies capable of directly binding and blocking (or ‘neutralising’) cell-free virus entry have been of great interest to vaccine developers and have ultimately been presented as the ‘holy grail’ of an effective vaccine. This view is understandable given the success of multiple vaccines that induce neutralising antibody responses, which are likely important for the control of infection (e.g., RSV, SARS-CoV-2, influenza). Indeed, the ongoing search for an HIV vaccine has long been founded on the identification of epitopes for broadly neutralising antibodies.

Consequently, the drive for vaccine-induced neutralising antibodies has also been predominant in strategies to develop HCMV vaccines by including AD-2 and AD-4 (known targets of neutralising antibodies) in gB-based vaccines, as well as, more recently, the addition of the pentameric complex. Again, this is not without precedent—studies have shown that AD-2 antibody responses are predictive of better outcomes following organ transplant [93] and in a maternal–foetal transfer setting [94].

However, over the past decade, the role of non-neutralising antibodies (which do not directly block or neutralise cell-free viral infection) and their protective capacity have been continuously highlighted by multiple research groups [95,96,97,98,99]. For example, these antibody responses can work by recruiting components of the immune system to infected cells (e.g., antibody-dependent cell cytotoxicity and NK cells, or complement) or can bind to pathogens to promote antibody-dependent phagocytosis by immune cells. The value of non-neutralising antibodies is becoming increasingly implicated in the context of vaccine development for other viral pathogens, including SARS-CoV-2, HIV, and influenza [100,101,102,103].

Thus, it became intriguing when, in two independent follow-up studies of sera from phase II gB/MF59 trials, no evidence for neutralising antibodies could be found. Notably, similar findings were reported using different methodologies, which are summarised in Table 3. The accumulating evidence strongly suggests that antibodies other than those that are directly neutralising against cell-free virus seem to play an undeniable role in eliciting protection against HCMV spread and disease.

Ultimately, both Nelson et al. and Baraniak et al. independently came to the same conclusion: the neutralising antibody titre elicited by gB/MF59 was not a reliable correlate of protection; however, the total antibody titre against gB was [105]. Thus, it was possible that the humoral protection elicited by gB/MF59 must be occurring, at least in part, via other antiviral mechanisms distinct from classical neutralisation.

Through further investigation, we observed variable vaccine-elicited responses against the classic immunological epitopes (AD-1 through AD-5) with no responses detected against AD-2 or AD-4 at all (against which potent neutralising antibodies are often directed) [95]. Moreover, whilst AD-1 and AD-5 responses were detected in some individuals, none could be correlated with protection. Likewise, Nelson et al. noted vaccine-induced antibodies poorly bound to known neutralising gB epitopes, particularly AD-1, AD-2 site 1, AD-5, and the combination of AD-5 with AD-4, compared to those from chronically infected individuals [98]. These mapping studies gave the first hints that the gB/MF59 vaccine was triggering an atypical humoral immune response compared to that observed in natural infection.

Of particular interest was when Nelson et al. analysed vaccine-induced antibody binding to linear gB epitopes, achieved by measuring responses to a peptide microarray library encompassing overlapping 15-mers that covered the whole Towne strain gB. Using this approach, vaccination appeared to predominantly elicit antibody binding to AD-3, present in 76% of vaccinated individuals versus 32% of naturally infected, with minimal response to the AD-2 site 1. Indeed, a similar analysis of the sera from vaccinated transplant patients also highlighted the presence of AD-3 responses in gB/MF59 vaccine recipient sera [83]. The potential paradox of a strong antibody inducer being found in (presumably) an antibody-inaccessible location raised many questions, with Nelson et al. presenting a hypothesis that it serves as one of the ‘decoy epitopes’ of HCMV—a concept that has also been postulated for AD-1 [106].

The consistent lack of robust responses against known gB antigenic domains in gB/MF59-vaccinated individuals pointed towards other gB epitopes being responsible for vaccine-induced immunity. As illustrated above in Figure 2, the Chiron gB has the furin cleavage site modified as well as substantial deletions aimed at removing the transmembrane domain to allow production as an extracellular protein. Hypothetically, different epitope presentation could be attributed to the modifications to the recombinant Chiron gB used in gB/MF59, potentially leading to the impaired folding of the molecule, resulting in a higher level of protomer-state molecules, different from the natural pre-fusion trimeric form of gB [33,92]. Could this be the explanation behind the vaccine-specific responses identified by Gomes et al. [83], where the immunogen allowed access to the normally hidden epitopes?

### 6.2. Does the Virus Hide Its Important Epitopes?

In 2023, a follow-up study of the gB/MF59 vaccine trial was conducted to further explore the role of non-neutralising antibody responses [83]. Sera samples from seronegative transplant patients were initially screened for binding affinity against a library of overlapping 15-mer peptides covering the entire gB protein. Subsequent analysis revealed distinct responses between vaccinees and the naturally infected seropositive population. As stated above, vaccinees exhibited robust responses against the AD-3 intracellular domain, as also reported by Nelson and colleagues in their study of the serum samples from the phase II trial in postpartum women [98]. However, the focus soon turned to the identification of responses directed against regions overlapping with structural domain V of gB, with the antigenic domain further termed AD-6. Considering these robust responses, further investigation of AD-6 was performed.

Remarkably, 78% of seronegative vaccinees showed a response against the AD-6 peptide, compared to less than 5% within the seropositive population. This provided one of the first hints towards a vaccine-specific response that, by extension, could underscore the potential importance of AD-6 responses in gB/MF59-vaccine-induced protection in organ transplant patients. An analysis of the phase II study on transplant patients noted that in the vaccinated D+R- subgroup, patients exhibited improved clinical outcomes if they had developed a response to AD-6. This suggested an antibody response against AD-6 as a potential correlate of protection—accepting all the caveats associated with retrospective analyses of phase II trial data. More fundamentally, this suggested that AD-6, although not sufficiently immunologically exposed in natural infection to induce antibody responses, was immunogenic when hypothetically more accessible in the vaccine context, probably due to the structural modifications within the Chiron gB showcased earlier in Figure 2. Interestingly, AD-6 antibody was shown to bind infected cells but not cell-free virions, which suggests that there might be a fundamental difference in epitope presentation between cell-associated and virion-associated gB.

Further analysis demonstrated that the AD-6 sequence is highly conserved across multiple strains of HCMV, showing 96.1% amino acid conservation and 99.98% pairwise identity. This conservation could suggest importance in gB function and an inability to tolerate substantial variation, which could be induced by immunological pressure. This immunological consideration would be consistent with our understanding of the functional importance of the structural domain V in gB function.

Immunological correlates of protection are crucial for the design, assessment, and implementation of vaccines. However, a major aspect of vaccine development is a desire to go beyond correlates of protection to mechanistic correlates of protection. Thus, we sought to understand whether there was any mechanistic basis for AD-6 as a potential protective response. A rabbit polyclonal antibody raised against AD-6 was shown to be non-neutralising against cell-free virus, consistent with the previous analyses on transplant patients’ sera discussed in the previous section [95,98]. Importantly, it was observed that the AD-6 pAb could bind to gB expressed on the surface of virally infected cells and that this binding had a functional impact—being able to block the cell-to-cell spread of a highly cell-associated strain of HCMV—confirming that antibodies against AD-6 could recognise (and be biologically active against) this region of gB in natural infection.

An outstanding question was whether the same is true for human antibodies. Notably, in the earlier 2018 study, Baraniak et al. noted that vaccine recipient sera had no overt antiviral properties and did not stop cell-cell spread [95]. However, a more granular inspection of the individual data suggested that effects were seen in some individuals; thus, this was re-visited with the caveat of using sera from the timepoint when the gB antibody titre was highest (after the second dose of the vaccine) rather than at the time of transplantation as performed in the original study. From these follow-up studies, it became clear that some vaccinee sera controlled infection spread, as opposed to sera from placebo patients. Importantly, in a number of cases (but not all), human AD-6 antibodies were responsible for the observed effect. Perhaps, more generally, what these data again demonstrated was that the binding to gB on the cell surface may be crucial to the activity of gB antibodies. Multiple studies from the Permar team have highlighted that IgG binding to plasma-membrane-associated gB alone is correlated with a decreased risk of acquiring HCMV [104]. Here, this binding is associated with enhanced antibody-dependent cell phagocytosis (ADCP) by macrophages. Importantly, the two events are not mutually exclusive, as both are reliant on antibody binding and strongly suggest that the targeting of gB in the context of cellular expression is important mechanistically for the activity of gB/MF59-induced gB-specific antibodies.

### 6.3. Are Neutralising Antibodies Against gB Not Important?

It remains an intriguing observation that the detection of neutralising antibodies was difficult in the analyses of the phase II gB/MF59 sera. Indeed, one is reminded that in the original phase I safety and immunogenicity studies, gB-neutralising antibodies were clearly detected, and their activity was enhanced by complement [107]. Furthermore, these data were corroborated by Nelson and colleagues using the same assays with which they had failed to detect neutralising antibodies from the phase II study sera [98]. An explanation for these differences in antibody activity between the phase I and phase II studies has remained elusive, although it is worth noting that neutralising antibodies were only robustly detected after the third dose of vaccination, which suggests that neutralising responses were difficult to induce. However, the phase I data did suggest it was possible for gB/MF59 to induce neutralising antibodies.

Thus, it was interesting to observe that in a follow-up study in the transplant cohort in 2019 [108], neutralising antibodies were rapidly evident post-transplant in the D+R- cohort. Specifically, we observed that 75% of the placebo group exhibited gB antibody levels below the threshold for the majority of their post-transplant period, only becoming detectable by day 90 post-transplant, whereas most vaccinated individuals showed greater IgG responses within 7 days of being challenged with HCMV (i.e., transplanted with an infected organ), which suggests that the gB/MF59 vaccine potentially had primed the immune system. In accordance with this, no IgM responses were noted when the sera were tested for antibodies directed against IE1, p150, pp65, and gB in placebo or vaccinees at days 7 and 35 post-transplant, which further suggests that no de novo responses were likely to have been made (consistent with the fact that these individuals are T-cell immune-suppressed, which would limit the production of new antibody responses). Further analysis revealed that the magnitude of these post-transplant gB-specific responses alone did not correlate with the outcome, unlike what was suggested in the earlier phase II study on transplant patients. This is perhaps easily explained by the fact that at a time post-transplant, the driver of gB-specific antibodies (in a prime boost model) would be viral replication, which is a clinical parameter directly correlated with poor outcomes. Thus, dissecting protective gB responses is inherently confounded in this specific situation.

More generally, it is important to highlight that the bigger picture of the gB/MF59 studies is that they do not suggest that neutralising antibodies are not going to be important for controlling the virus but rather that they do not solely determine the infection outcome. Indeed, returning to the studies of the phase I sera where neutralisation was observed, it was also demonstrated that the presence and durability of neutralising antibodies were very time-dependent [107]. One intriguing hypothesis, therefore, is that gB/MF59 can induce antibodies with neutralising activity, but they are not durable. However, a clear memory recall response may be possible even in post-transplant immune-suppressed patients [108].

Further evidence that would be partially consistent with the hypothesis that the vaccine primes the immune system with undetectable neutralising responses in the long term was supported by a follow-up study of pre-transplant patient sera from the phase II study [83]. Focusing on the same high-risk D+R- transplant subpopulation, neutralisation assays were performed in an analogous way to previous studies, complemented by a modified neutralisation protocol that aimed to detect responses not previously identified. In this modified neutralisation assay, fibroblasts were infected with HCMV at 4 °C to prevent viral entry while allowing the virus to bind to the surface, potentially triggering the gB conformational changes necessary for fusion and entry and, concomitantly, using temperature to select for the potential of low-level yet high-affinity neutralising antibodies, which can compete much better for their targets at lower temperatures. Using this approach, the presence of neutralising antibodies was revealed in the phase II sera. Although in the context of transplantation (performed at 37 °C), the physiological relevance is unclear, these data provided proof of principle that antibodies with the capacity to neutralise HCMV were present after the vaccination. Defining what epitopes those neutralising antibodies recognise will be highly instructive and, hypothetically, indicate the targets of the monoclonal antibodies identified in the prior analysis of the phase I sera discussed above [107].

The precise importance of gB-neutralising antibodies for a successful vaccine against HCMV is a highly nuanced conversation. Clearly, the success of the gB/MF59 vaccine cannot be attributed to neutralising antibodies, but, again, we reiterate that this does not rule out that neutralising antibodies will have an important role to play in the context of vaccination against HCMV. We are reminded that in the HCMV-seropositive patients vaccinated with gB/MF59, there was a clear boost to anti-AD-2 antibody levels if prior immunity to AD-2 was present in those individuals before vaccination. Furthermore, the presence of an anti-AD-2 response was ultimately correlated with a better outcome post-transplant, which was also observed in an earlier study of renal transplant recipients [109] and in models of congenital transmission [94]. Pre-clinical studies of AD-2-specific monoclonal antibodies (e.g., TRL345) have proven promising; however, peptide vaccines based on AD-2 have failed to induce the potent neutralising responses we observe in naturally infected people, which has led to a stall in the development of AD-2-based approaches [75,110].

We also highlight that the expression of the gB ectodomain (similar to the antigen presentation in gB/MF59) anchored to the VSV-G scaffold in virus-like particles has demonstrated a capacity to induce neutralising antibodies in a recent phase I safety study [61]. One can hypothesise that this is because, in this case, gB is being presented to the immune system as a pre-fusion homotrimer, and, therefore, better represents its native virion state. Having a similar approach in mind, multiple groups have demonstrated the struggle to stabilise the pre-fusion gB as a recombinant protein but have also reported its ability to elicit immune responses better than its post-fusion counterpart [111,112]. Indeed, it is hypothesised that achieving the ‘perfect’ pre-fusion form of gB is going to be a game-changer for HCMV vaccination, as this will induce the ‘desired’ gB antibodies.

## 7. Other Major Trials

Although this review has focused on gB/MF59—which is the HCMV vaccine that has arguably been investigated the most in follow-up studies—it is important to highlight that there are other vaccine candidates that have shown promising results so far and are, thus, currently being investigated in ongoing phase II and III trials.

One such vaccine is Moderna’s mRNA-1647, delivering mRNA constructs encoding for full-length gB plus all five components of PC. It has shown an acceptable safety and immunogenicity profile in its recently concluded phase I clinical trial [68]. Importantly, mRNA-1647 elicited both humoral and cellular immune responses within the healthy adult population of phase I trial participants. While the vaccine induced gB-specific antibody responses in the HCMV-seronegative population, they did not reach the benchmark levels demonstrated by the seropositive vaccinees. The same was not observed for PC-specific antibody titres, with vaccinated seronegative participants developing a robust and long-lasting response that surpassed the seropositive benchmark. Moreover, some of the protective effect was hypothesised to be linked to the increased level of ADCC activity exhibited by the vaccine recipient sera [113]. It has been previously reported that gB is a poor ADCC target, which may suggest PC-specific antibodies as being responsible for the observed effect [114]. Importantly, the resultant total neutralising antibody titres were further replicated and confirmed in a separate phase II dose-finding clinical trial, which resulted in the progression of the vaccine to phase III [115]. The results of an ongoing phase III of the mRNA-1647 trial are imminent in 2025, and so, the relative success of this approach at controlling HCMV infection is eagerly anticipated.

In the context of this review and gB, it is interesting to note that the preliminary studies on mRNA-1647 vaccine sera from earlier studies demonstrated that the generated gB-specific responses were somewhat different when compared to those elicited by the gB/MF59 vaccine [113]. Of note, they reported that there is no AD-6 reactivity and reduced ADCP activity—two immune responses considered to be important for the success of gB/MF59 [83,98].

This variation in responses between both vaccines containing gB further emphasises the importance of understanding how gB is presented to the immune system; in the mRNA vaccine, full-length gB mRNA is translated by the cell, likely presenting gB in its native full-length form—different from the modified gB present in the gB/MF59 vaccine. Perhaps, if future vaccine efforts should aim to mimic the protective gB/MF59 responses, then it is essential to consider how gB will be presented so that critical epitopes are accessible. Indeed, the ‘plug and play’ aspects of mRNA lend themselves ideally to this approach. As stated, 2025 is when we are likely to know the relative success of the mRNA-1647 vaccine, and it remains possible that the inclusion of pentamer with native gB is going to be sufficient using the RNA platform.

A similar strategy of enhancing PC and gB antigen presentation was applied to the vaccine candidate called V160, which was recently tested in clinical trials.

V160 is a conditionally replication-defective virus (based on the AD169 strain, which has had the PC complex repaired) whose life cycle depends on Shield-1, a synthetic compound essential for growing and propagating V160 in vitro [116]. Similarly to mRNA-1647, V160 was demonstrated to elicit antibody responses against gB, PC, and also pp65, as well as additional robust T-cell response against IE1 and pp65 in healthy adult participants of a phase I clinical trial [103,117]. Subsequently, post hoc analyses of a phase II clinical trial of V160 demonstrated 42.4% efficacy (32–50% depending on the endpoint chosen) [118]. However, similarly to other virion-based vaccines like Towne, V160 failed to significantly reduce HCMV acquisition by the seronegative vaccinees. This raises the question of whether primary viral acquisition is at all preventable by any vaccination strategy and, perhaps, whether the new generation of HCMV vaccines should focus on preventing disease rather than infection and consider this an acceptable endpoint for HCMV vaccine evaluation.

## 8. Discussion and Future Directions

The gB/MF59 vaccine has demonstrated superior efficacy even when compared to other vaccines containing gB alongside other HCMV antigens. This superior efficacy implies that it triggers distinct immune responses, underscoring the significance of understanding not only the vaccine components but also how they are presented to the immune system. Recent research efforts have concentrated on identifying these crucial immune responses, initially pointing to gB antibody titre as a correlate of protection, and directing enquiries towards understanding the underlying mechanisms [95].

Historically, vaccine efficiency is usually attributed to neutralising antibody titres, an assumption initially made for the HCMV vaccine as well. Recent studies of the gB/MF59 vaccine have challenged this view and suggest that other components of humoral immunity could be equally, if not more, important.

However, while the neutralising antibody titre was not identified as a correlate of protection in gB/MF59 vaccine studies, it is essential not to disregard the potential protective role of neutralising antibodies and the possibility of enhancing vaccine efficacy by adding new HCMV components (or presenting gB differently) to increase neutralisation responses. This ‘cumulative approach’ underpins the logic of multi-epitope vaccines, like the aforementioned mRNA-1647 and V160 vaccines.

The detection of distinct vaccine responses between gB/MF59 and Moderna’s mRNA-1647 raises interesting questions regarding the potential effectiveness of the gB component of the mRNA-1647 vaccine. If it fails to trigger the protective gB responses identified in gB/MF59 studies, is it possible that mRNA-1647 is relying largely on anti-PC neutralising responses [113]? Considering that PC deletion from Rhesus CMV in a Rhesus macaque model did not impact cCMV transmission, this may suggest that pentamer responses may not have an effect on this transmission route and, thus, that success via PC will rely on preventing transmission to the mother [119]. With that being said, one must always remain cautious of over-interpreting animal models with respect to vaccine development and pathogenesis, and the answer will come once the data from the phase III study are made available.

Another important biological consideration is that neutralising antibodies are often unable to inhibit cell-to-cell spread (assumed to be the mode by which cCMV infection occurs in vivo), potentially due to limited access to the targets where this mode of transmission occurs [120]. More generally, it reminds the reader about HCMV’s biphasic modes of growth (cell–cell spread and cell-free spread), suggesting that an effective vaccine will likely have to elicit various antibody effector functions to control both. Therefore, whilst there is a place for an effective vaccine to also neutralise cell-free HCMV, gB/MF59 studies have highlighted that non-neutralising antibody functions must not be neglected in future formulations. Indeed, returning to animal models, it was very nicely demonstrated that non-neutralising antibodies can control HCMV replication in a mouse model of transplantation, which illustrates this point [121].

The newly emerging “plug and play” vaccine technologies like mRNA offer the rapid development of multi-epitope vaccines for testing in non-human models. This allows for the combination of various gB epitopes—for example, the addition of AD-6, the removal of AD-3, or the incorporation of different HCMV components like pp65 and the gH/gL/gO trimer—or even more recently identified targets for inducing potent ADCC responses, including UL16 and UL141 [114]. With this technology, the path to a comprehensive HCMV vaccine has the potential to become significantly more streamlined, offering the ability to rapidly prototype and iterate vaccine designs based on emerging basic research and clinical trial data—and, crucially, allowing investigators to precisely define the immune responses they seek to achieve in vaccinated individuals. Going forward, a major consideration is understanding the precise nature of antibody recognition against the specific epitopes we desire. For example, is targeting a specific linear sequence (e.g., AD-2) or a conformational epitope in HCMV (e.g., AD-5 or AD-6) going to be more desirable? Given the challenges regarding the proper induction of conformation-specific antibodies, a viable alternative could be to utilise delivery vectors like virus-like particles engineered to display multiple epitopes and present antigens in specific conformations, which could allow for a more controlled and fixed gB (or any desired) presentation.

Due to the focus of this review being on gB/MF59, which has been investigated largely from a humoral immunity perspective, it is critical to remember the undeniable importance of T-cell immunity, which should not be overlooked when considering the new generation of HCMV vaccines. Many older HCMV vaccines included IE1 and pp65, which are immune-dominant in the T-cell response following natural infection [122]. Cellular immunity is critical for the control of HCMV, and, thus, identifying important targets for T-cell-mediated killing will make an important contribution to the design of a future vaccine [123,124,125]. Notably, an induction of CD4+ and CD8+ T-cell immunity has been a key criterion before in HCMV vaccine development, with multiple vaccine candidates setting it out as a desired criterion for vaccine efficacy evaluation [27]. Indeed, additional value to HCMV vaccine development can be brought from novel computational methods of predicting plausible T-cell epitopes [126]. It is easy to argue that a perfect HCMV vaccine should combine in itself the ability to induce a robust T-cell response, as well as neutralising and non-neutralising antibody responses.

Moving from vaccine composition determination to implementation is the next critical step. Deciding whom to immunise and what endpoint will define success remain open questions, though scientists and public health officials have put forward various strategies. When considering markers of success, key considerations include whether the vaccine should reduce viral spread from toddlers to mothers, prevent infection of mothers altogether, block cCMV transmission, and/or reduce disease in congenitally infected infants. Because pre-existing HCMV immunity does not completely prevent reinfection or reactivation (but limits pathogenesis and disease), aiming for sterilising immunity is often viewed as an unrealistic target by many. Instead, focusing on the reduction of cCMV transmission and the severity of disease—achieved by reducing the infection of pregnant women or limiting the cross-placental transfer of HCMV—may be more feasible. gB/MF59 studies have focused on the ability of the vaccine to stop maternal infections; however, gB-based vaccines in guinea pigs have shown to be more effective in preventing cCMV rather than maternal infection, which justifies the clinical evaluation of current and future vaccine candidates to prevent cCMV transmission and disease rather than maternal infection [127]. The clear limitation or barrier for such an approach in human trials is the likely numbers required to generate statistically significant evidence of vaccine effectiveness—hence, why clinical studies of maternal infection (or in transplant patients) are more common—outlining the pragmatist’s balance between desired versus measurable clinical outcomes.

Drawing parallels with the SARS-CoV-2 vaccine, which primarily halts disease progression and reduces transmission through lower viral loads, a similar strategy could be used for HCMV. This could involve immunising children to cut transmission at its most common source or women of childbearing age to prevent foetal transmission/disease. However, societal acceptance poses a dilemma. The reluctance observed during the SARS-CoV-2 vaccination efforts, particularly towards vaccinating children against a disease with no major effects on them, could portend potential hesitancies in adopting a similar approach for HCMV. However, vaccinating women of childbearing age emerges as an alternative, motivated by the desire of mothers to protect future offspring. Yet again, a substantial number of women also have apprehensions about vaccine safety during or near pregnancy, which suggests that targeting adolescent females could be a more effective strategy, ensuring immunity is established well before pregnancy risks arise. However, this then raises the question of the durability of the vaccine-elicited immune response and, specifically, how long the vaccine protection lasts.

Research on the gB/MF59 vaccine has mainly targeted seronegative individuals, leaving its potential to enhance natural immunity against reactivation/superinfection less explored. Preliminary studies, however, show the vaccine’s ability to enhance the immune response in those already infected, particularly by boosting gB CD4+ T-cell- and AD-2-specific responses [93,128]. Notably, in seropositive transplant recipients, the gB/MF59 vaccine amplified pre-existing AD-2 responses, correlating with a lower incidence of viraemia. This also raises an additional consideration: given that a significant portion of the population, especially women of childbearing age, is already seropositive for HCMV, there is a pressing need to optimise vaccines to prevent not just primary infection but also reactivation and superinfection, therefore reducing cCMV acquisition rates. Given that the gB/MF59 HCMV vaccine studies in seropositives suggest that the vaccine only boosts pre-existing immune responses, future vaccines will have to be designed to present novel antigens, as seropositives’ immune systems have already encountered and developed responses to the virus’s existing antigens [108]. Here again, the plug-and-play approach of mRNA vaccine technology may become useful and may lead to different vaccines based on HCMV serostatus.

Altogether, studies of the gB/MF59 vaccine candidate have shown promise in the development of an effective HCMV vaccine. They represent a shift away from conventional assumptions regarding the indispensability of neutralising antibodies for successful HCMV vaccine development, instead outlining the need for a multifaceted approach. Importantly, these studies highlighted that identifying protective immune responses against HCMV induced by vaccines—regardless of their abundance during natural infection—could be of great importance towards our collective goal of identifying the immunological ‘Achilles heel’ of a virus that is a specialist at immune evasion. This only serves to further emphasise the need for future investigations to not only precisely target and enhance these protective responses but also identify novel protective responses. Importantly, lessons learnt from HCMV vaccine development could potentially translate into strategies for addressing other latent viruses like HIV, for which an effective vaccine is also yet to be developed but is so urgently desired.

## 9. Conclusions

The development of an effective HCMV vaccine has been of the highest priority for several decades; however, there is no licensed vaccine at the moment of writing this review. Analysing the more successful vaccine candidates of the past, such as gB/MF59, may reveal the mechanistic correlates of vaccine-induced protection and, thus, aid in the development of the new generations of HCMV vaccines. Conversely, also understanding why certain vaccines were less effective could also be informative (albeit harder to decipher). Understanding which aspects of the large and broad immune response are particularly crucial for the control of HCMV are clearly going to be critical—and these could likely include immune responses rarely induced during natural infection that have been revealed in vaccination studies.

## Figures and Tables

**Table 1:** Summary of HCMV vaccine candidates based on a limited number of specific HCMV antigens. NA is not applicable (i.e., Phase I study).

Vaccine Name (Company/Developer)	Antigen(s)	Antigen Presentation/Delivery	Most Advanced Clinical Trial Stage	Reported Efficacy for Protection(for Phase II and Up)	Reference(s)
gB/MF59 **(Sanofi Pasteur and Chiron, now Novartis—adjuvant)**	gB	Modified (Chiron) gB—mutated furin cleavage site and removed transmembrane domain. Adjuvanted with MF59	Phase II	43–50%	[55,56,57]
GSK1492903A **(GSK**)	gB	Chimeric HCMV gB fused with HSV-1 gD	Phase I	NA	[58,59]
ASP0113 (VCL-CB01) **(Astellas Pharma and Vical)**	gB, pp65	Two plasmids that encode HCMV pp65 and gB adjuvanted with poloxamer CRL1005 and benzalkonium chloride	Phase III	Exact number not reported. Not better than placebo	[60]
VBI-1501A **(VBI Vaccines)**	gB	Enveloped virus-like particle expressing HCMV gB modified to have VSV G transmembrane domain	Phase I	NA	[61]
ALVAC-pp65 **(Sanofi Pasteur)**	pp65	Replication-deficient canarypox vector	Phase II	Number not reported	[62]
ALVAC-gB **(Sanofi Pasteur)**	gB	Replication-deficient canarypox vector	Phase I	NA	[62]
AVX601 **(AlphaVax)**	gB, pp65, IE1	Aphavirus replicon particle expressing gB and a pp65-IE1 fusion protein	Phase I	NA	[63]
Triplex **(Helocyte)**	pp65, IE1, IE2	Poxvirus vector (MVA) expressing pp65, IE1-exon4, and IE2-exon5	Phase II (ongoing)	To be reported	[64]
HB-101 **(Hoopkia)**	gB, pp65	Two replication-deficient choriomeningitis viruses expressing gB and pp65	Phase II (ongoing)	To be reported	[65]
CMVPepVac **(Renal Center Heidelberg)**	pp65	HCMV pp65 peptide with Aldara adjuvant	Phase I	NA	[66]
CMVPepVax **(City of Hope and The National Cancer Institute)**	pp65	Chimeric peptide consisting of CD8+ T-cell epitope of pp65 and a tetanus T-helper epitope	Phase Ib	NA	[67]
mRNA-1647 **(Moderna)**	gB and PC	mRNAs encoding for gB and five proteins comprising PC within lipid nanoparticles	Phases II and III (both ongoing)	To be reported	[68]
HCMV SAM **(Novartis Vaccines and Diagnostics)**	gB, pp65, IE1	Self-amplifying RNA encoding for gB and pp65-IE1 fusion protein in a cationic nanoemulsion	Phase I	NA	[69]

**Table 2:** Overview of the different phase II gB/MF59 HCMV vaccine clinical trial protocols. All trials adopted a randomised, double-blind, placebo-controlled approach, focusing on different interest groups: postpartum women, adolescent women, and solid-organ transplant patients. Measured objective (bold) and methodology are shown.

Trials	Target Population	Age	Vaccination Schedule	Time of Follow-Up After First Vaccination	Measured Objective(s) + Method	gB/MF59 Efficacy
[57]	Seronegative postpartum women	14–40	Vaccine or placebo at 0, 1, and 6 months	42 months	**Rate of seroconversion:** testing for IgG against non-gB HCMV proteins, further confirmed with q-RT PCR.	50%
[55]	Seronegative adolescent girls	12–17	Vaccine or placebo at 0, 1, and 6 months	34 months	**Rate of seroconversion:** gB-adsorption assay followed by sera analysis **Viral shedding:** q-RT PCR on various body fluid samples **gB IgG levels:** ELISA	43–45%
[56]	Seronegative and seropositive solid-organ transplant recipients	50.5 (mean age)	Vaccine or placebo at 0, 1, and 6 months	Up to 13 months	**Presence of HCMV DNA in blood:** q-RT PCR **gB IgG levels:** ELISA **Neutralising antibody levels:** neutralisation assay using human fibroblasts	50%

**Table 3:** Comparison of methods used in Nelson et al. and Baraniak et al. to investigate the composition of the antibody response in gB/MF59 phase II trials.

References	Study Analysed	Neutralisation Measured How?	Other Antiviral Activity Measured?	Conclusions
[98]	Phase I trial in healthy adults	Direct neutralisation against autologous (Towne) and heterologous (AD169, TB40e) strains	Neutralising ability with the addition of complement. Later studies also explored ADCP, ADCC, and NK cell degranulation.	Superior neutralisation ability (with and without the complement) when compared to the sera from phase II trial on postpartum women, possibly due to physiological differences influenced by pregnancy and childbirth [104]
	Phase II trial in postpartum women			Limited neutralising ability pre- and post-vaccination against heterologous strains, slight increase in neutralising ability against Towne. Possible ADCP and ADCC mechanism of protection. Total titre of gB-specific antibodies is the correlate of protection, despite limited neutralising titre.
[95]	Phase II trial in transplant patients	Direct neutralisation against clinical Merlin strain.	Neutralising ability with the addition of complement. Also explored NK cell degranulation.	Limited neutralising ability pre- and post-vaccination against Merlin with and without the addition of the complement. No effect of sera on NK cell degranulation. Limited influence of ADCC on vaccine-induced protection. Total titre of gB-specific antibodies is the correlate of protection, despite limited neutralising titre.

## Abbreviations

The following abbreviations are used in this manuscript:

AD	Antigenic Domain
EBV	Epstein–Barr Virus
ELISA	Enzyme-Linked Immunosorbent Assay
HCMV	Human Cytomegalovirus
HIV	Human Immunodeficiency Virus
HSV-1	Herpes Simplex Virus
IgG	Immunoglobulin G
IgM	Immunoglobulin M
FL	Fusion Loop
FCS	Furin Cleavage Site
PC	Pentameric Complex
UK	United Kingdom
US	United States
VSV	Vesicular Stomatitis Virus
